# The role of respiratory viruses in the etiology of bacterial pneumonia: An ecological perspective

**DOI:** 10.1093/emph/eow007

**Published:** 2016-02-15

**Authors:** Kyu Han Lee, Aubree Gordon, Betsy Foxman

**Affiliations:** ^1^Department of Epidemiology, School of Public Health, University of Michigan, 1415 Washington Heights, Ann Arbor, MI 48109, USA

**Keywords:** co-infection, ecology, pneumonia, influenza, *Streptococcus pneumoniae*

## Abstract

Pneumonia is the leading cause of death among children less than 5 years old worldwide. A wide range of viral, bacterial and fungal agents can cause pneumonia: although viruses are the most common etiologic agent, the severity of clinical symptoms associated with bacterial pneumonia and increasing antibiotic resistance makes bacterial pneumonia a major public health concern. Bacterial pneumonia can follow upper respiratory viral infection and complicate lower respiratory viral infection. Secondary bacterial pneumonia is a major cause of influenza-related deaths. In this review, we evaluate the following hypotheses: (i) respiratory viruses influence the etiology of pneumonia by altering bacterial community structure in the upper respiratory tract (URT) and (ii) respiratory viruses promote or inhibit colonization of the lower respiratory tract (LRT) by certain bacterial species residing in the URT. We conducted a systematic review of the literature to examine temporal associations between respiratory viruses and bacteria and a targeted review to identify potential mechanisms of interactions. We conclude that viruses both alter the bacterial community in the URT and promote bacterial colonization of the LRT. However, it is uncertain whether changes in the URT bacterial community play a substantial role in pneumonia etiology. The exception is *Streptococcus pneumoniae* where a strong link between viral co-infection, increased carriage and pneumococcal pneumonia has been established.

## INTRODUCTION

Pneumonia is the leading cause of death in children <5 years worldwide, which is responsible for one million deaths each year [1]. The burden is greatest in developing countries, at an estimated 0.22 episodes per child-year, but remains a major public health concern even among developed countries where there are an estimated 0.015 episodes per child-year [2]. In the USA, pneumonia is the second only to newborn infant births as the most common reason for hospital admissions (36 cases per 10 000 persons [3]) and causes nearly 50 000 deaths each year [4].

A wide range of viral, bacterial and fungal agents can cause pneumonia when aspirated into the lungs. The Centers for Disease Control and Prevention Etiology of Pneumonia in the Community (EPIC) study identified viruses as the most commonly identified etiologic agent in children and adults hospitalized with pneumonia. An etiologic agent was detected in 81% of 2222 children <18 years of age: 66% had one or more viral pathogens, 8% one or more bacterial pathogens and 7% both bacterial and viral pathogens [5]. Among 2259 adults, an etiologic agent was detected in 38%: 23% had one or more viral pathogens, 11% one or more bacterial pathogens and 3% both bacterial and viral pathogens [6]. However, virtually all these serious pneumonia cases were treated with antibiotics, as secondary bacterial infection can complicate lower respiratory viral infection. Therefore, even in cases determined to be pneumonia solely of viral etiology, bacterial interactions of virus and bacteria may play some role.

Explanatory Box 1. Challenges in determining the etiology of pneumonia

Even in countries where pneumonia surveillance is routinely conducted such as the USA, no information on microbial etiology is recorded for approximately 65–85% of hospitalized pneumonia cases [11, 12]. Severely ill patients often are not included in surveillance, organisms on the causal pathway may have been cleared by the time that the patient presents clinically or prior to testing because of rapid treatment with antibiotics when pneumonia is suspected, and autopsies are infrequently done on the elderly. To optimally determine etiology, direct sampling via bronchoalveolar lavage is required, but usually detection of causal agents is conducted on blood, sputum and urine because of ease of collection, ethical issues and costs. Bacteremia is observed in only 7%–13% of adult pneumonia cases and 1–5% in child pneumonia cases, sputum can potentially be contaminated by bacteria in the URT and is difficult to obtain from children, and blood and urine antigen assays require further validation or are limited to adults and specific to only a few pathogens (e.g. *Streptococcus pneumoniae* and *Legionella* species) (reviewed by Murdoch *et al.* [13]). Although modern molecular biologic techniques make it feasible to conduct untargeted screens for all bacterial, viral and fungal species present, it is still difficult to distinguish between infection, colonization or contamination [14]. Continued efforts are needed to develop more accurate methods to determine the etiology of pneumonia, and thus maximize treatment and prevention efforts.

The large proportion of pneumonia cases without a detected pathogen underscores the limitations of current surveillance and detection methods and how they frame our understanding of pneumonia etiology (Box 1). EPIC study results suggest we may not be detecting the full panel of pathogens in cases we currently define as viral pneumonia nor considering the potential role of bacteria on the pathogenic potential of viruses. Bacterial causes of pneumonia are associated with more severe clinical symptoms and increasing antibiotic resistance complicates treatment [2, 7–10], making bacterial causes of pneumonia a major concern.

In this review, we examine two hypotheses that argue the etiology of bacterial pneumonia is a consequence of ecologic selection influenced by the interaction of respiratory viruses and bacteria within the host: (i) respiratory viruses influence the etiology of pneumonia by altering bacterial carriage structure in the upper respiratory tract (URT) and (ii) respiratory viruses promote or inhibit colonization of the lower respiratory tract (LRT) by certain bacterial species residing in the URT. We begin by describing the normal processes of bacterial selection in the upper and LRTs and then present evidence on how these processes can potentially be altered by respiratory viruses.

## METHODS

We conducted a systematic literature search in PubMed for studies published between 1 January 1990 and 9 December 2015. We restricted studies to those conducted in the USA to minimize potential geographic variation of associations. The following search string was used: ‘(bacteria[All Fields] OR bacterial[All Fields]) AND (virus[All Fields] OR viral[All Fields]) AND (lower respiratory tract infection[All Fields] OR LRTI[All Fields] OR lower respiratory tract[All Fields] OR LRT[All Fields] OR lower respiratory infection[All Fields] OR LRI[All Fields] OR pneumonia[All Fields] OR bronchitis[All Fields]) AND (“1990/01/1”[PDAT]: “2015/12/09”[PDAT]) AND United States[All Fields] AND (time[All Fields] OR temporal[All Fields] OR season*[All Fields])’*.* Among 464 articles written in English, exclusions were made based on titles, abstracts and full articles. We excluded reviews, *in vivo* and *in vitro* experiments, and studies of immunocompromised populations. Nine articles were retrieved from the literature search and three additional studies were selected from the reference list of retrieved articles.

## BACTERIAL SELECTION IN THE URT

Bacterial pneumonia is primarily caused by the commensal bacteria normally residing in the URT [11, 12]. The most common causes of bacterial pneumonia for children <5 years of age are *Streptococcus pneumoniae,* followed by *Haemophilus influenzae* and *Staphylococcus aureus* [11] although this varies over time and space. From a rudimentary ecological perspective, the human respiratory tract can be defined as an ecosystem with two distinct niches: the URT, characterized by regular asymptomatic carriage of commensal bacteria, and the LRT, which is inhabited at a low abundance by bacteria in healthy individuals [13]. During the first year after birth, the nasopharynx is rapidly colonized [14] and URT carriage is established via ongoing synergistic and antagonistic interactions among commensal bacteria [15]. Although pneumonia is an infection of the lungs, microbial selection in the URT may play an important role in etiology as bacterial strains in the URT can be readily aspirated into the LRT. For example, URT carriage is believed to be a necessary precursor of pneumonia due to *S. pneumoniae* [16, 17].

Numerous epidemiologic studies describe synergistic and antagonistic relationships among various commensal bacteria [18–33] and, although the exact biological mechanisms remain unclear, *in vivo* and *in vitro* experiments suggest potential mechanisms involve either direct interaction between bacterial species or indirect interactions via the host immune system (Table 1). A number of population studies suggest that *S. pneumoniae* carriage is positively associated with *H. influenzae* [18–27] and *Moraxella catarrhalis* [18, 23–28] carriage but negatively associated with *S. aureus* [19, 20, 24–27, 29–32]. Furthermore, *S. aureus* carriage is generally negatively associated with *H. influenzae* and *M. catarrhalis* [19, 24, 31] carriage, whereas *H. influenzae* and *M. catarrhalis* are believed to be positively associated [18, 22, 24, 33]. Nevertheless, our understanding is limited, as the dynamics of niche competition likely consist of complex relationships between multiple species [31, 34] and strains [15, 35], further influenced by host and environmental factors [22, 36]. As carriage is an important precursor of respiratory infections for certain bacterial species [12], unraveling the complex system of bacterial interactions that determine URT microbiota may be key factor for understanding the etiology of pneumonia.

**Table 1:** Known interactions and potential mechanisms for observed associations between primary bacterial colonizers of the nasopharynx

Organism 1	Organism 2	Interaction^a^	Potential mechanisms^b^
*S. pneumoniae*	*S. aureus*	Antagonism [19, 20, 24–27, 29–32]	Hydrogen peroxide production [107]
			Catalase [108]
			Pilus [109]
			Immune-mediated competition [110, 111]
*S. pneumoniae*	*H. influenzae*	Synergism [18–27, 112]	Provision of nutrients [15]
			Production of β-lactamase [113]
			Formation of biofilms [113]
			Phosphorychlorine expression [12]
*S. pneumoniae*	*H. influenzae*	Antagonism [31]	Hydrogen peroxide production [114]
			Catalase [114]
			Desialylation [115]
			Immune-mediated competition [15, 116, 117]
*S. pneumoniae*	*M. catarrhalis*	Synergism [18, 23–28]	Passive antibiotic protection [118, 119]
*S. pneumoniae*	*M. catarrhalis*	Antagonism	Hydrogen peroxide production [114]
*S. aureus*	*H. influenzae*	Synergism [22]	Provision of nutrients [15]
*S. aureus*	*H. influenzae*	Antagonism [19, 24, 31]	
*S. aureus*	*M. catarrhalis*	Antagonism [24]	
*H. influenzae*	*M. catarrhalis*	Synergism [18, 22, 24, 33]	Outer membrane vesicles [120]

a Epidemiologic studies.

b *In vitro* and *in vivo* experiments

## BACTERIAL SELECTION IN THE LRT

Lung microbiome studies suggest that bacteria colonizing the LRT overlap with those found in the URT, but that the abundance of organisms is quite low [13], and their role in pneumonia etiology has yet to be explored. To colonize the LRT, an organism must overcome mucociliary clearance and phagocytosis by resident alveolar macrophages, neutrophils and monocyte-derived macrophages [37, 38], but many URT pathogens have developed strategies to overcome these barriers. *H. influenzae*, *Mycoplasma pneumoniae* and *Bordetella pertussis* resist mucociliary clearance by impairing ciliary function. *Streptococcus pyogenes, Streptococcus agalactiae, H. influenzae, Neisseria meningitidis* and *S. pneumoniae* possess capsules that resist phagocytosis [37]*. Streptococcus pneumoniae*, the leading cause of pneumonia [39], is characterized by over 90 serotypes differentiated by variations in the bacterial polysaccharide capsule [40, 41] and associated with different propensities of invasive potential [42]. In addition to protecting against phagocytosis, the capsule prevents clearance by mucous secretion and restricts autolysis [43]. Other species, including *S. aureus,* release anti-opsonizing proteins and possess surface protein A to evade phagocytosis. Furthermore, *S. aureus* secretes leukotoxins that lyse leukocytes and express superantigens that hinder immune response (reviewed by Naber *et al.* [44]).

The crucial role these various mechanisms play in determining respiratory disease is demonstrated by contrasting *M. catarrhalis* with *S. pneumoniae.* Similar to *S. pneumoniae*, *M. catarrhalis* is a primary carriage species estimated to colonize between 31% and 50% of children <2 years in the USA [45] and frequently causes URT infections, such as acute otitis media. However, unlike *S. pneumoniae*, *M. catarrhalis* rarely causes pneumonia [46], suggesting that differences in mechanisms of pathogenicity may be the explanation.

## RISK FACTORS OF BACTERIAL PNEUMONIA

**Figure 1.**
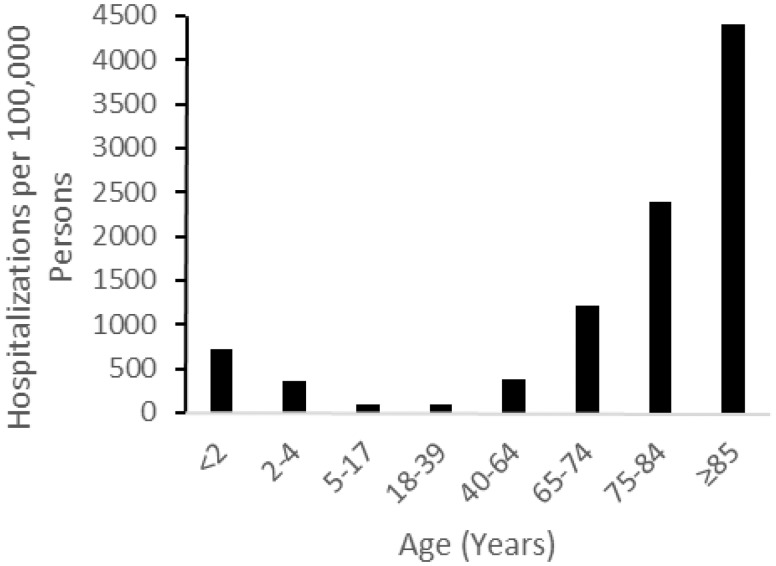
Rate of hospitalization for pneumonia; the USA, 2007–2009. Adapted from Griffin *et al.* [48]

Various other factors—including underlying medical conditions and smoking—can increase the risk of pneumonia by compromising pulmonary clearance mechanisms and the host immune response [47], potentially influencing the selection of pathogens in both the URT and LRT . Age plays a major role in pneumonia risk. In developed countries, such as the USA, the risk of pneumonia is highest in individuals who are 65 years or over (Fig. 1) [48]. The elevated risk in the elderly is likely due to impaired host defenses and an increase in comorbidities—heart failure, liver disease and underlying lung disease—which increase risk of aspiration pneumonia that can occur from dysphagia and gastroesophageal reflux disease (reviewed by Akgün *et al.* [49]). In developing countries, the burden of pneumonia is greatest in young children [2] due to their inability to physically remove and immunologically deal with bacterial pathogens (reviewed by Siegrist [50]). Very young children also have the greatest prevalence in the nasopharynx of common bacterial pneumonia pathogens: *S. pneumoniae*, *H. influenzae* and *M. catarrhalis* [23, 51]. Increased carriage may be an important risk factor for pneumonia if the URT bacterial community structure is a determinant of pneumonia etiology. Unfortunately, the majority of carriage studies have been conducted among children <5 years of age, which limits our ability to establish the role of nasopharyngeal carriage in other age groups.

Regardless of age, viral infection is an important risk factor for bacterial pneumonia. Viruses can lead to rapid, drastic increases in morbidity and mortality in all age groups as seen in historic influenza epidemics and pandemics [52], making it a major public health concern.

## TEMPORAL ASSOCIATIONS BETWEEN VIRUSES AND BACTERIA

The 1918 Spanish flu pandemic resulted in ∼50 million deaths worldwide: most of the deaths were caused by secondary bacterial pneumonia [53, 54]. During the 2009 H1N1 pandemic, bacterial co-infection was detected in 18–34% of influenza cases (reviewed by Chertow and Memoli [8]) with vulnerability peaking ∼1 week after influenza infection [55]. The association of viral infection and bacterial pneumonia is not limited to influenza although that interaction has been most studied: adenovirus, human metapneumovirus, respiratory syncytial virus (RSV), and other viruses have been temporally associated with an increased risk of pneumococcal pneumonia and invasive pneumococcal disease (IPD), defined as the isolation of *S. pneumoniae* from a normally sterile site, in the USA (Table 2) [56–67]. The majority of US studies suggest strong associations between *S. pneumoniae* infections (both pneumonia and IPD) and influenza virus and RSV, with potential effect modification by age. Temporal associations with other viruses are less supported and limited to IPD. We did not find any studies in the USA that examined temporal associations between viruses and bacterial species other than *S. pneumoniae*. Six studies conducted in other developed countries examined temporal associations between respiratory viruses and IPD [68–73]. Three out of five studies that examined influenza virus found associations with IPD in UK, The Netherlands and Sweden [70, 71, 73]. Among four studies that examined RSV in other countries, two indicated associations with IPD in all age groups [70, 71], one found an association only among children [68] and the last observed an association only in individuals 2 years or older [69].

**Table 2:** Temporal associations between respiratory viruses and *S. pneumoniae*, the USA

Study	Virus	Outcome	Age group	Temporal association
Kim *et al.* [56]	ADV	IPD	All	Yes
	IV			Yes
	PCV			No
	PIV			No
	RSV			Yes
	All			Yes
	except			
	IV			
Talbot *et al.* [57]	IV	IPD	All	Yes
	RSV			Yes
Ampofo *et al.* [58]	ADV	IPD	<18 years	No
	hMPV			Yes
	IV			Yes
	PIV			No
	RSV			Yes
Murdoch and Jennings [59]	IV	IPD	All	Yes
	PIV1			No
	PIV2			No
	PIV3			Yes
	RSV			Yes, only in < 5 years
Nelson *et al.* [60]	IV	IPD	All	Yes
Walter *et al.* [61]	IV	Pneumonia	All	Yes
Zhou *et al.* [62]	IV	Pneumonia	All	Varies by season
	RSV			Varies by season
Weinberger *et al.* 2012 [63]	2009 H1N1 season	Pneumonia	All	Yes
Shrestha *et al.* [64]	Influenza seasons	Pneumonia	All	Yes
Fleming-Dutra *et al.* [65]	2009 H1N1 season	Pneumonia	All	Yes
Weinberger *et al.* 2014 [66]	RSV	Pneumonia	<7 years	Yes
Weinberger *et al.* 2015 [67]	RSV	Pneumonia	<1 years	Yes
			1 to < 2 years	Yes
	IV		<1 years	No
			1 to < 2 years	Yes

Abbreviations: ADV (adenovirus), hMPV (human metapneumovirus), IV (influenza virus) and PCV (picornavirus)

Temporal associations provide evidence of virus–bacterial interactions, but do not necessarily prove these interactions exist. Many viral infections are seasonal, as is pneumonia infection, so the temporal associations may merely reflect the influence of other seasonal phenomena, environmental or host, that are shared by both viral infection and pneumonia [74]. However, evidence for true virus–bacterial interactions is supported by population studies that estimate a high prevalence of viral co-infection during pneumonia [5, 6] and animal models that suggest increased susceptibility to pneumonia and increased disease severity during viral co-infection [75]. In the USA, ∼47% of children and 19% of adults with bacterial pneumonia are co-infected with one or more viruses [5, 6]. Further, vaccination for *S. pneumoniae* reduced pneumonia associated with RSV, influenza A and parainfluenza (PIV) types 1-3 [76]. Influenza vaccine probe studies may provide additional insight to the burden of influenza co-infection on bacterial pneumonia.

## RESPIRATORY VIRUS ALTERS ASYMPTOMATIC CARRIAGE OF KNOWN BACTERIAL PATHOGENS

**Figure 2.**
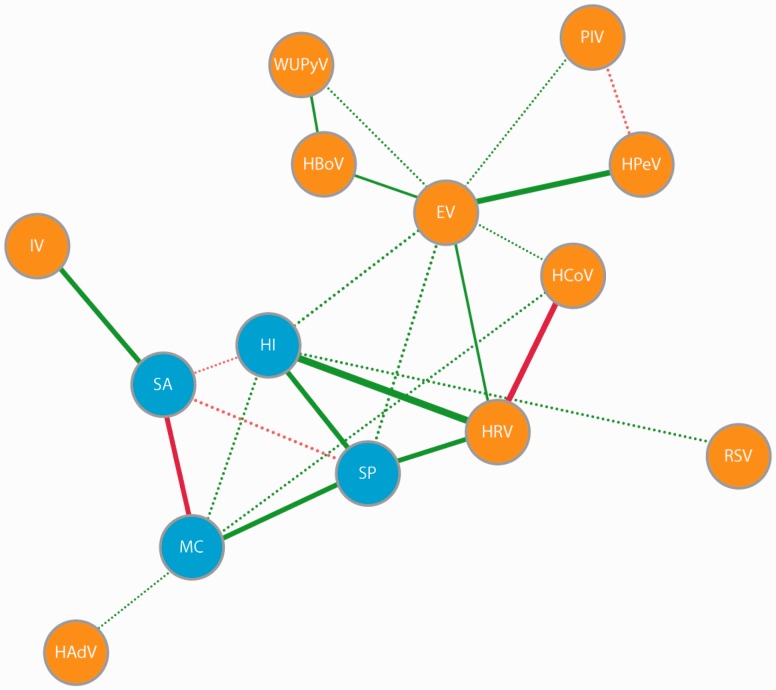
Network of interactions between virus and bacteria in the upper respiratory tract. Figure 1A in van den Bergh *et al.* [26] used under the Creative Commons Attribution License. Green lines indicate synergistic associations and red lines indicate antagonistic associations. Solid lines indicate associations with *P* < 0.01 and dashed lines indicate associations with *P* between 0.01 and 0.05 for associations between species. enterovirus (EV), *H. influenzae* (HI), human adenovirus human (HAdV), bocavirus (HBoV), human coronavirus (HCov), human parechovirus (HPeV), human rhinovirus (HRV), influenza virus (IV), *M. catarrhalis* (MC), *S. aureus* (SA), *S. pneumoniae* (SP) and WU polyomavirus (WUPyV)

Consistent with our first hypothesis, viral infection frequently has been associated with carriage of common pneumonia pathogens. In a cross-sectional analysis of aboriginal and non-aboriginal children in Western Australia, Jacoby *et al.* [18] observed positive associations between rhinovirus and *S. pneumoniae*, *H. influenzae* and *M. catarrhalis* and a positive association between adenovirus and *M. catarrhalis* in the nasopharynx. In a US study, children with a viral URT co-infection not associated with otitis media had a higher prevalence of nontypeable *H. influenzae* and *M. catarrhalis* relative to healthy children. Furthermore, children with viral co-infection associated with acute otitis media had an increased prevalence of *S. pneumoniae*, nontypeable *H. influenzae* and *M. catarrhalis* but a decreased prevalence of α-hemolytic *Streptococci* [77]. van den Bergh *et al.* [26] assessed the prevalence of 20 respiratory viruses and the main commensal bacteria in the nasopharynx of 433 healthy Dutch children aged 6–24 months. In their study, rhinovirus was positively associated with *S. pneumoniae* and *H. influenzae*, RSV was positively associated with *H. influenzae*, coronaviruses and adenovirus were positively associated with *M. catarrhalis*, and influenza virus was positively associated *S. aureus* (Fig. 2). However, as the associations found in the above-mentioned studies are based on cross-sectional analyses, we cannot determine whether viruses influenced carriage structure, bacterial carriage influenced host susceptibility to viruses or if bidirectional interactions occurred. Prospective studies are required to resolve this temporal ambiguity.

Although the impact of the host microbiota on viral infections is an important consideration (reviewed by Wilks *et al.* [78]), the majority of *in vivo* experiments pertaining to virus–bacterial interactions in the URT focus on the role of viruses on the host microbiota. The results of these studies suggest that viruses can alter carriage structure by promoting the colonization of certain commensals. In both animal models and human adults, infection with influenza A virus showed increased colonization by *S. pneumoniae* and *H. influenzae* in the URT [79–83]. Similarly, infecting rats and chinchillas with RSV led to increased colonization by nontypeable *H. influenzae* [84, 85]. Collectively, epidemiologic studies and laboratory experiments suggest that the introduction of a virus to the URT niche can substantially alter the bacterial community present [26].

## THE MISSING LINK BETWEEN BACTERIAL CARRIAGE STRUCTURE AND PNEUMONIA

Although there is substantial evidence that viral infection influences the URT bacterial community, whether these changes are reflected in the LRT and ultimately in pneumonia etiology is unclear, which weakens our first hypothesis (i.e. respiratory viruses can influence the etiology of pneumonia by altering bacterial carriage structure in the URT). Studies that examine the joint effects of viral co-infection, bacterial carriage and bacterial pneumonia would provide one strategy for filling this gap. However, we found only two such studies. In a South African hospital-based surveillance study of severe acute respiratory illness, 969 nasopharyngeal-oropharyngeal specimens were tested for *S. pneumoniae* and a panel of respiratory viruses. A high pneumococcal colonization density in the nasopharynx and oropharynx was associated with both respiratory virus co-infection and pneumococcal pneumonia [86]. A second hospital-based case–control study compared nasopharyngeal carriage among 274 radiologically confirmed cases of pneumonia, 276 cases of other LRT infections and 350 controls in Vietnam. Their findings for *S. pneumoniae* were similar to that of the South African study. However, the investigators also studied *H. influenzae* and *M. catarrhalis* and found no clear association between viral co-infection, nasopharyngeal bacterial load and pneumonia for these species [87]. As noted above, *M. catarrhalis* rarely causes pneumonia, but *H. influenzae* is second only to *S. pneumoniae*. Although there appears to be a persuasive argument for a link between viral co-infection, carriage and pneumonia for *S. pneumoniae*, whether or why the interaction is not true for other URT bacteria needs further exploration. In particular, studies that can directly test whether viral infection led to bacterial colonization or overgrowth by a potential pathogen, which led to bacterial pneumonia by that pathogen, are in order. In conclusion, there is no definitive answer to our first hypothesis. Epidemiologic studies and experiments indicate viruses alter the bacterial community in the URT, but they do not yet adequately address whether these changes in the URT bacterial community play a significant role in pneumonia etiology.

## MECHANISMS OF INTERACTION SUGGEST THAT VIRUS CAN ALTER BACTERIAL SELECTION IN THE LRT

There are several studies that support our second hypothesis, that respiratory viruses can promote bacterial colonization of the LRT by certain commensals in the URT. Viruses interact with bacteria and the host at various stages along the pathologic pathway to promote bacterial pneumonia (Table 3). For example, virus can increase shedding of URT bacteria into the LRT: *in vitro* biofilm and murine studies suggest influenza A virus infection can lead to the dispersion of *S. pneumoniae* biofilms, releasing virulent pneumococci for subsequent secondary infections in the LRT [88, 89]. When in a biofilm, *S. pneumoniae* is less virulent; capsule polysaccharide and pneumolysin production are reduced and synthesis of the bacterial adhesin phosphorylcholine increased [90, 91].

**Table 3:** Mechanisms of synergistic virus-bacteria Interaction

Mechanism	Virus	Bacteria
Biofilm dispersion	IAV	*S. pneumoniae* [88, 89]
Increased expression of cell surface receptors	ADV	*S. pneumoniae* [121]
	IAV	*S. pneumoniae* [122]
	PIV	*H. influenzae* [100, 123]
	RSV	*S. pneumoniae* [100, 123]
		*H. influenzae* [100, 123]
		*S. pneumoniae* [100, 123]
Direct binding of virus and bacteria	RSV	*S. pneumoniae* [124, 125]
Damaged and inhibited repair of respiratory epithelium cells	IAV	*S. aureus* [26]
		*S. pneumoniae* [75]
Decreased mucociliary velocity	IAV	*S. pneumoniae* [127]
Viral neuraminidase	IAV	*S. pneumoniae* [95, 96]
Impairment of leukocytes (i.e. neutrophils) response	IAV	*S. aureus* [128]
	RSV	*S. pneumoniae* [104, 105, 129, 130]
		*S. pneumoniae* [131]
Impairment of alveolar macrophage response	IAV	*S. aureus* [101, 132–134]
Impairment of monocytes	IAV	*S. aureus* [128]
	RSV	*M. catarrhalis* [135]
		*NTHi* [135]
		*S. pneumoniae* [135]
Reduced natural killer cell recruitment	IAV	*S. aureus* [136]
Exacerbation of inflammatory mediators and tissue damage	hMPV	*S. pneumoniae* [137]
	IAV	*S. pneumoniae* [138–141]

Abbreviations: ADV (adenovirus), IAV (influenza A virus), hMPV (human metapneumovirus), NTHi (nontypeable *H. influenzae*), PIV (parainfluenza virus), and RSV (respiratory syncytial virus).

Viral infections also can promote bacterial adhesion to host cells [92–94]. Influenza and PIV promote bacterial adhesion with respiratory epithelium cells by cleaving sialic acid and exposing receptors on host cell oligosaccharide chains [95, 96]. *In vitro* and *in vivo* experiments suggest free sialic acid released by viral neuraminidase can behave as signaling molecules promoting pneumococcal biofilm formation, nasopharyngeal colonization and bacterial spread to the lungs [97]. Free sialic acid is believed to play a role in invasion by nontypeable *H. influenza* as it is an important component of the biofilm matrix and incorporated into the bacterial capsular polysaccharide to evade host defense mechanisms [98]. Although literature is scarce, the relationship may be bilateral as bacterial neuraminidase can promote virus survival during treatment with neuraminidase inhibitors [99]. In addition, viruses can promote bacterial adhesion by upregulating cell surface receptors for pathogenic bacteria. For example, RSV and PIV-3 infection can lead to upregulation of receptors intracellular adhesion molecule 1 (ICAM-1), carcinoembryonic antigen-related cell adhesion molecule 1 (CEACAM1), and platelet-activating factor receptor (PAF-r) to promote binding of nontypeable *H. influen*zae and *S. pneumoniae* to epithelial cells [100].

Respiratory viral infection can damage and impede the repair of respiratory epithelial cells leading to reduced mucociliary clearance. Consequently, bacteria can more easily enter the lungs to cause pneumonia [75]. Many of the virus–bacteria interaction mechanisms involve viral compromise of the innate immune system. These include impairment and depletion of resident alveolar macrophages [101–103] and neutrophils, which are necessary for bacterial clearance, mediated by induction of type I interferons [104] and desensitization to Toll-like receptor ligands [105]. Detailed descriptions of potential biological pathways involved in these mechanisms are discussed in earlier reviews by Robinson *et al.* [55] and McCullers [9]. Finally, excessive inflammation in the lungs due to virus-initiated exacerbation of inflammatory mediators, cytokines and chemokines, can cause tissue damage [6], which increases susceptibility to secondary bacterial infections.

Despite the considerable literature on potential mechanisms of viral–bacterial interactions that may lead to pneumonia, most studies are limited to experiments conducted in animal models using select viral and bacterial strains, which may not reflect what is occurring in human populations. Furthermore, the interactions between virus and bacteria are undoubtedly far more complex than identified in animal models, and likely consists of a complex web of interactions between different viruses and bacteria with viruses similar to that described in the URT [12, 26]. Even after considering these limitations, the overwhelming evidence for the existence of multiple biological mechanisms under various conditions supports our second hypothesis that respiratory viruses can alter bacterial selection in the LRT and is an important factor in pneumonia etiology.

## CONCLUSIONS

In this review, we discussed how the respiratory tract is an ecosystem with two niches, the URT and the LRT; each with ecological and microbial pressures that determine bacterial selection. We hypothesized that viruses influence bacterial selection in the URT leading to colonization of the LRT and sometimes pneumonia. There appears to be a complex network of interactions among viruses and bacteria in the URT that responds to viral introduction by altering what bacteria are present or modifying their relative abundance. For a least one species, *S. pneumoniae,* viruses can increase nasopharyngeal carriage density and increase risk of pneumococcal pneumonia. Whether this is true for other URT bacteria that cause pneumonia is uncertain. We also proposed that bacterial selection in the LRT could be altered by viral infection. The LRT is normally inhabited by low density of microbes, a state maintained by local host defenses and bacterial mechanisms of evasion. *In vitro* and *in vivo* studies suggest viruses can promote entry and colonization of the LRT for select bacterial species via a range of biological mechanisms including URT biofilm dispersion, increased bacterial adhesion to host epithelial cell by upregulation of cell receptors, reduced pulmonary clearance, impairment of multiple components of the innate immune response and changes in inflammatory response. Although there are limitations in interpreting the results of experiments, evidence of numerous mechanisms observed under various conditions strongly suggest that viruses also play an important role in the selection of bacteria in the LRT and pneumonia etiology.

The greatest difficulty in addressing our hypotheses was our inability to determine the relative contributions of URT bacterial community structure and local host defenses on bacterial selection into the LRT. In the simplest case, how much is the risk of pneumonia following viral infection attributable to the presence of a known bacterial pneumonia pathogen (such as *S. pneumoniae*) in the URT? To determine this, studies must examine time-dependent carriage of bacteria, species-specific pneumonia outcomes and the effects of viral co-infection among other known risk factors—which, to the best of our knowledge, do not currently exist. Nonetheless, the literature strongly supports the presence of an interaction between viral infection and secondary bacterial pneumonia; the failure to fully understand the mechanisms should act as a spur for future studies while continuing current efforts to reduce the worldwide burden of pneumonia. 

## CONFLICTS OF INTEREST

Dr. Gordon has received consultancy fees from Abt Associates. All other authors report no potential conflicts of interest.
